# Translational Rodent Models for Research on Parasitic Protozoa—A Review of Confounders and Possibilities

**DOI:** 10.3389/fcimb.2017.00238

**Published:** 2017-06-07

**Authors:** Totta Ehret, Francesca Torelli, Christian Klotz, Amy B. Pedersen, Frank Seeber

**Affiliations:** ^1^FG16 – Mycotic and Parasitic Agents and Mycobacteria, Robert Koch InstituteBerlin, Germany; ^2^Department of Molecular Parasitology, Humboldt-Universität zu BerlinBerlin, Germany; ^3^School of Biological Sciences, University of EdinburghEdinburgh, United Kingdom

**Keywords:** wild rodent, protozoa, parasite, model organism, mouse, rat, translational research

## Abstract

Rodents, in particular *Mus musculus*, have a long and invaluable history as models for human diseases in biomedical research, although their translational value has been challenged in a number of cases. We provide some examples in which rodents have been suboptimal as models for human biology and discuss confounders which influence experiments and may explain some of the misleading results. Infections of rodents with protozoan parasites are no exception in requiring close consideration upon model choice. We focus on the significant differences between inbred, outbred and wild animals, and the importance of factors such as microbiota, which are gaining attention as crucial variables in infection experiments. Frequently, mouse or rat models are chosen for convenience, e.g., availability in the institution rather than on an unbiased evaluation of whether they provide the answer to a given question. Apart from a general discussion on translational success or failure, we provide examples where infections with single-celled parasites in a chosen lab rodent gave contradictory or misleading results, and when possible discuss the reason for this. We present emerging alternatives to traditional rodent models, such as humanized mice and organoid primary cell cultures. So-called recombinant inbred strains such as the Collaborative Cross collection are also a potential solution for certain challenges. In addition, we emphasize the advantages of using wild rodents for certain immunological, ecological, and/or behavioral questions. The experimental challenges (e.g., availability of species-specific reagents) that come with the use of such non-model systems are also discussed. Our intention is to foster critical judgment of both traditional and newly available translational rodent models for research on parasitic protozoa that can complement the existing mouse and rat models.

## Introduction

Gregor Mendel introduced the basic concept of a “model organism” when he reported his experiments on plant hybrids. He picked peas as a model because they had clear experimental advantages for addressing his question: “*At the very outset special attention was devoted to the* Leguminosae *on account of their peculiar floral structure (…) this led to the result that the genus* Pisum *was found to possess the necessary qualifications*.” (Mendel, 1866) Since then this approach for model selection for a particular purpose is widely used, meaning that a model organism should be accessible, experimentally tractable, have short generation times, be affordable to maintain and breed, possess clearly identifiable features that are to be studied, and more recently be genetically tractable (which includes access to a sequenced genome), to name a few (Rand, 2008). Given the importance of model systems in biology, the history and diversity of model organisms has been extensively reviewed (Conn, 2008; Hunter, 2008; Müller and Grossniklaus, 2010; Ankeny and Leonelli, 2011; Bolker, 2012; Alfred and Baldwin, 2015).

The available model organisms span a great taxonomic range, and for many questions single-celled organisms such as bacteria (*Escherichia coli*) or yeasts (*Saccharomyces cerevisiae*) are sufficient. However, in many biomedical studies which aim to translate findings to humans, non-mammals are not applicable as translational models (Hau, 2008). Yet, shorter phylogenetic distances and anatomical similarities are no guarantee for translational success, as research on primates has demonstrated (detailed in section The CD28 Superagonist Antibody “Disaster”). Given the high relevance of protozoa for human and animal health and our own scientific interests in these parasites, we will concentrate on such infections in rodents. While their value as translational models is not without dispute (see section Protozoan Parasites) they have been, still are, and will continue to be, invaluable for both basic biological questions in host-parasite interactions as well as pre-clinical studies. Their importance for scientific progress is demonstrated, for example by the essential role laboratory mice played in the discovery of dendritic cells (see references in Steinman, 2012) and macrophages (reviewed by Gordon, 2007). The value of rodents, and mice in particular, has also been highlighted for leading to fundamental insights in infection biology (Buer and Balling, 2003; Vidal et al., 2008; Douam et al., 2015). Given the historical and current importance of rodents, we here explore benefits and limitations of, e.g., inbred or outbred mice, lab rodents, or rodents from the wild, etc., as well as possible external confounders, such as breeding conditions in lab facilities or interactions in an ecosystem, that might have great impact on research results. With this review, we encourage experimentalists, particularly in translational medicine, to consider a broad set of potential rodent models in order to identify and use the best available system for specific studies. We aim to provide a foundation and useful references for such decisions.

## Protozoan parasites

Infections with protozoan parasites cause substantial illness and economic loss in humans worldwide (see Tables 1, 2 for details; Fletcher et al., 2012; Murray et al., 2012; Andrews et al., 2014; Robertson et al., 2014; Kassebaum et al., 2016). These parasites with high impact on humans mostly are the Amoeba, e.g., *Entamoeba* spp.; the flagellates, e.g., *Trichomonas* spp., *Giardia* spp., *Leishmania* spp., and *Trypanosoma* spp., and the large group of Apicomplexans, which contain, e.g., *Plasmodium* spp., *Toxoplasma gondii*, and *Cryptosporidium* spp. They are most often transmitted to their host either via ingestion of contaminated food, water, or via a vector (e.g., mosquitoes or flies; see Table 2). Here we provide a brief overview of some parasitic protozoa which substantially impact humans, and of which many are referred to in our examples used in following sections.

**Table 1:** Global “Disability-Adjusted Life-Years,” DALYs, for high impact infectious diseases, with several examples from protozoan parasites.

	DALYs (^*^1,000) 2010^a^	DALYs (^*^1,000) 2015^b^
Lower respiratory infections	115,227 (95,983)^c^	142,384
Influenza	19,244	nr^d^
Diarrhoeal diseases	89,513 (78,904)^e^	84,928
HIV/AIDS	81,547	62,759
Tuberculosis	49,396	56,037
Protozoan diseases, total	97,884 (15,199)^f^	40,695
Malaria	82,685	38,520
Leishmaniasis	3,317	1,357
African trypanosomiasis	560	372
Chagas disease	546	253
Trichomoniasis	167	194
Cryptosporidiosis	8,372	nr^d^
Amoebiasis	2,237	nr^d^

*Data shown for 2010 and 2015. For causative agents of protozoan diseases see Table 2*.

a *Murray et al. (2012)*.

b *Kassebaum et al. (2016)*.

c *Number in brackets, without influenza*.

d *nr, not reported*.

e *Number in brackets, without cryptosporidiosis and amoebiasis*.

f *Number in brackets, without malaria*.

**Table 2:** The diseases caused, transmission routes, and suitability of rodents as models for human disease are listed for selected protozoan parasites.

**Parasite species**	**Disease (organ(s) mainly affected)**	**Transmission route**	**Suitability of rodent model for human infectious species**	**References**
*Plasmodium* spp.	Malaria (blood and liver)	Vector	(yes)	Cowman et al., 2016
*Toxoplasma gondii*	Acute and congenital toxoplasmosis (brain, heart, systemic)	Food, water, congenital	yes	Schluter et al., 2014
*Cryptosporidium* spp.	Cryptosporidiosis (intestine)	Food, water	(yes)	Checkley et al., 2015
*Trichomonas vaginalis*	Trichomoniasis (urogenital tract)	Sexual	(yes)	Kusdian and Gould, 2014
*Giardia duodenalis*	Giardiasis (intestine)	Food, water	(yes)	Ankarklev et al., 2010
*Entamoeba* spp.	Amoebiasis (intestine, liver, other organs)	Food, water	(yes)	Stanley, 2003
*Leishmania* spp.	Cutaneous and visceral leishmaniasis (skin; several organs)	Vector	(yes)	Stuart et al., 2008; Akhoundi et al., 2016
*Trypanosoma brucei (gambiense* and *rhodesiense)*	African trypanosomiasis/sleeping sickness (blood, lymphatics, brain)	Vector	(yes)	Matthews, 2015
*Trypanosoma cruzi*	Chagas disease (heart, systemic)	Vector	(yes)	Messenger et al., 2015

*Reference is given to a single article describing the basic biology of the respective protozoan to serve as starting point for further reading*.

*(yes), adopted to model*.

The significance of protozoan infections for global human health is here exemplified by data (see Table 1) where the impact of infections by the most devastating protozoan parasites is expressed as “disability-adjusted life-years” (DALYs, used by the World Health Organization (WHO) and others as a measure of disease impact). These diseases ranked second in importance across all infectious diseases, behind lower respiratory infections, and before AIDS and tuberculosis. The great majority of this impact can be attributed to malaria alone (85% caused by *Plasmodium* spp.). However, the collective disease burden of the other protozoa evaluated was also substantial and in the range of influenza (Murray et al., 2012). While many of these figures, including those for malaria, are fortunately on the decline, this disease was still ranked among the top 20 leading diseases as identified by the WHO worldwide in 2015 (Kassebaum et al., 2016). In addition to these human health concerns, protozoan parasites cause significant losses in many species of domestic animals (Perry and Grace, 2009; Torgerson and Macpherson, 2011; Fitzpatrick, 2013; Torgerson, 2013) and are in some cases a conservation concern for wildlife (Pedersen et al., 2007).

While many of these parasites have a very restricted host range (infecting a single host species and/or tissue), other extremes such as *T. gondii*, a zoonotic parasite assumed to infect all nucleated cells in all warm-blooded animals, exist. Consequently, there is a mixture of more or less natural relationships between the parasites and the rodent hosts when the latter are used as translational models for human infections (see Table 2). Many parasite genera contain species which naturally infect rodents (e.g., *Plasmodium, Giardia, Cryptosporidium*), although in most cases the very same species do not also infect humans. For other parasite species, the rodent model has been made susceptible, frequently by genetic means, to human relevant parasites (e.g., *P. falciparum* or *C. parvum/C. hominis*). In malaria research, rodents have been successfully used as models, but the suitability of the mouse to mimic severe human malaria has been questioned (Langhorne et al., 2011). In leishmaniasis research, rodents are acknowledged for contributing to a better understanding of the immune response to the parasite (Lipoldová and Demant, 2006) but other authors point out limitations and the lack of suitability of certain mouse strains to study specific parasite genotypes (Mears et al., 2015). Research on human sleeping sickness (*T. brucei*) has benefitted largely from mouse models (Antoine-Moussiaux et al., 2008; Giroud et al., 2009; Magez and Caljon, 2011) but criticism has been raised that more suitable animal models should be applied to address sleeping sickness in livestock (*T. congolense* and *T. vivax;* Morrison et al., 2016).

## Why are mice and rats such popular models?

**Figure 1:**
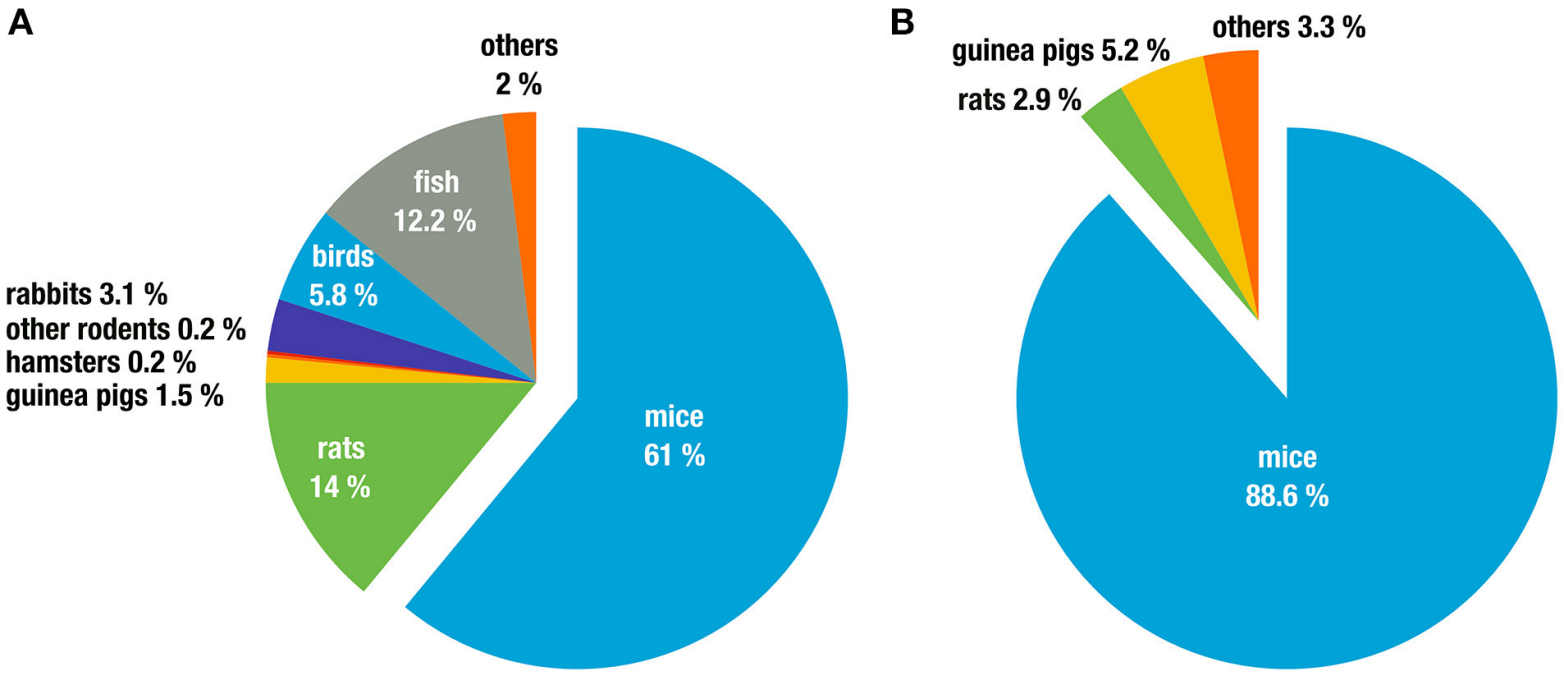
Animal classes used in experiments for **(A)** all life sciences disciplines in the 27 EU member states (2011 in %). Data taken from (The Commission to the Council and the European Parliament, 2013), and **(B)** research on infection biology only, in Germany (2011 in %). Data taken from (German Federal Ministry of Food and Agriculture, 2011).

Biomedical research depends heavily on model organisms and the majority of these are rodents, particularly in infectious disease and immunological research. A few numbers illustrate this impressively. For example, in the European Union alone, 75% of all animals used for “experimental and other scientific purposes” in 2011 were house mice (*Mus* spp. 61%) and rats (*Rattus* spp. 14%; The Commission to the Council and the European Parliament, 2013). Other rodents (gerbils, hamsters, different species of mice, and other rodents) only constitute 0.47% of animals used (Figure 1A). In Germany, 91.5% of animals used in research on infectious diseases were rodents, with the vast majority being *Mus musculus* (88.6%; Figure 1B). Similar numbers are reported in the United Kingdom, with 82% of all research using rodents, again dominated by *M. musculus* (74.6%; UK Home Office, 2015). While these numbers also include animals that were used as donors, e.g., for blood or organs and thus for *in vitro* experimentation these data nevertheless illustrate the dominance and importance of rodents, in particular laboratory inbred mice, as model organisms.

**Figure 2:**
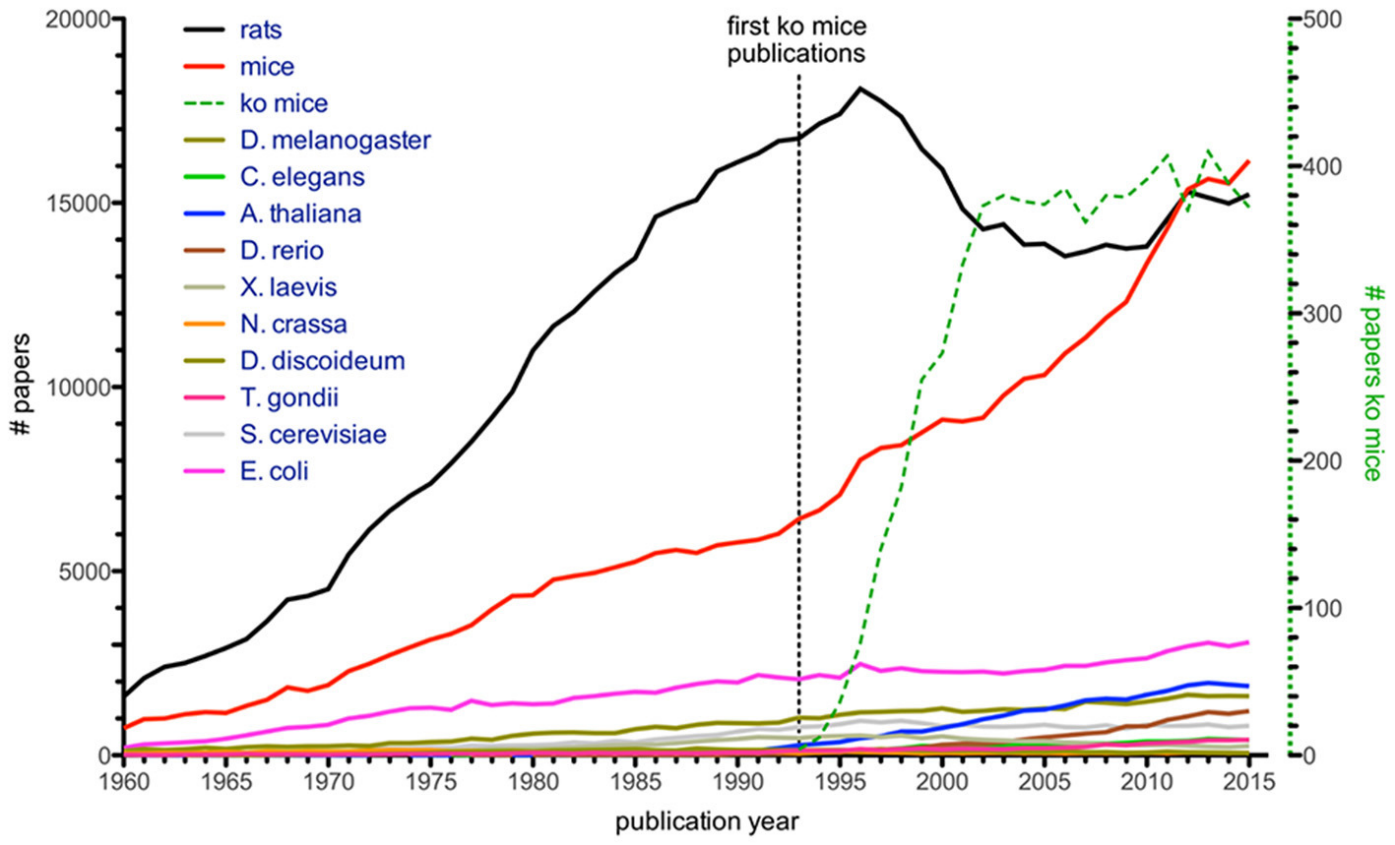
Number of citations with organism's name in title (Dietrich et al., 2014), based on Web of Science entries for a given year. Numbers for single-celled model organisms like *T. gondii, S. cerevisiae*, and *E. coli* are given for comparison. Green dashed line (with corresponding y-axis on the right) illustrates articles mentioning knock-out mice, with first papers appearing in the early 1990s.

Another informative figure shows that the number of publications where mice and rats were mentioned in the title dominates all other model organisms included (Figure 2), e.g., *Arabidopsis, Drosophila, S. cerevisiae, Caenorhabditis elegans, Xenopus*, zebrafish, *Neurospora*, and *Dictyostelium discoideum*. It is presumably no coincidence that a sharp increase in these “mouse publications” was seen in the 1990s, given that it was when embryonic stem cell manipulation met homologous recombination of the mouse genome. This resulted in the generation of defined gene knock-out mice (Figure 2), a finding which was later rewarded with the Nobel Prize in Physiology (Mak, 2007). The importance of this discovery for scientific progress in infection biology cannot be overestimated. However, in rats no such methods were available until relatively recently (Tong et al., 2010; van Boxtel and Cuppen, 2010), which is reflected in the drastic increase of mouse models and a relatively stable use of rats from 1990s until now. It is likely due to the highly developed genetic tools in mice, together with the more than 450 inbred mouse strains established since the first strain (DBA/2) was developed by Clarence Cook Little, that mice, and in particular the C57BL/6 strain, are the most popular animal model (Beck et al., 2000; Festing and Fisher, 2000). However, with the advent of CRISPR/Cas9 gene modifications in rats this will likely change (Hu et al., 2013; Li D. et al., 2013; Li W. et al., 2013), since this method has worked so far in almost all organisms tried and it can most likely also be applied to wild rodents. The historic establishment of tools for mice combined with the fact that 99% of genes are conserved between the human and the mouse genomes (Waterston et al., 2002) has made and will continue to make the mouse an obvious choice for translational efforts, i.e., research to understand the basics of and find treatments for human diseases. In addition, it will be exciting to see contributions from so far poorly explored model systems.

### The mice we use in experiments — who are they and how do they live?

Here we will briefly cover definitions of nomenclature for referring to different types of mice, from laboratory to “wild” rodents.

**Classical inbred mice** are defined as either being “produced using at least 20 consecutive generations of sister x brother or parent x offspring matings” or “traceable to a single ancestral pair in the 20th or subsequent generation.” (“Nomenclature of Inbred Mice,” defined by the Mouse Genomic Nomenclature Committee). However, it can be noted that 20 generations of inbreeding does not lead to fixed alleles in the entire genome, although for most phenotypes no differences are detected after this threshold (Chia et al., 2005). Different inbred populations exist and are referred to as strains (whereas outbred populations are often referred to as stocks). Inbred strains are genetically highly homogenous, well-defined, and often with genomes and SNP data available. In addition, extensive descriptions of (mutant) strains are available in the Mouse Phenome Database (Grubb et al., 2014) or the International Mouse Phenotyping Consortium database (Koscielny et al., 2014; see Table S1 for links) and should be consulted when planning experiments.

**Wild-derived inbred** strains are “descendants of mice captured in wild populations during the mid to late 20th century and represent several different *Mus* species from around the world” (Lutz et al., 2012). These mice are considered suitable for, e.g., evolutionary studies and gene mapping, but notably do not represent the genetic diversity of wild animals.

**Outbred** stocks are defined as “a closed population (for at least four generations) of genetically variable animals that is bred to maintain maximum heterozygosity” (Chia et al., 2005), meaning that each individual is genetically different from the others. Once established, the goal is to keep the genetic variability between generations to a minimum which is achieved by using, e.g., a certain number of breeding pairs (Chia et al., 2005). We onwards refer to inbred and outbred *M. musculus* models as lab mice, if nothing else is specified.

**Recombinant Inbred Strains**, RIS, are a collection of mice established by inbreeding two existing inbred strains into a set of strains (often called set or panel). Each such strain is genetically homogenous, but “parallel” strains produced from the same two well-defined ancestral strains are genetically more different from each other than either of the two ancestors (Chia et al., 2005). One advantage of using a RIS set compared to pure inbred strains is that phenotypic differences (e.g., pathogen or drug susceptibility) can be fairly easily assigned to specific genotypes (Guénet et al., 2015), and obtaining high-quality quantitative data on transcripts and proteins is feasible (Chick et al., 2016). Other options for lab mice, such as genetic crosses, will be covered in the section Humanized Mice: Rodents Which Mimic the Human Immune System.

We refer to **wild rodents** (including wild mice, e.g., species of *Mus*) as rodents which breed without direct intervention or manipulation by humans, in their natural habitats, e.g., farmland, forests or cities (Singleton and Krebs, 2007). Such populations may in some cases be under experimental study and manipulated, for example by regular trapping, diet manipulations and drug treatment, and will here still be considered as wild populations.

### Mouse housing influences experimental outcome

Almost all animal research facilities can house rodents in specific pathogen-free, SPF, barrier facilities. This standard includes regular screening for a large set of common pathogens (in order to detect contamination), and commonly autoclaving cages, bedding, water, food and other housing related materials to assure hygienic and controlled housing, as well as controlled light/dark cycles (Hedrich and Nicklas, 2012). For details see respective lab manuals (Ayadi et al., 2011; Hedrich, 2012). Animal psychological status has been shown to influence variability in experimental studies, including examples of more reproducible results from “happier” mice, which display less anxiety or depression-associated behavior as a result of increased animal welfare (Bayne and Würbel, 2012). Although, wild mice can run several 100 m per night (Latham and Mason, 2004) including a means for physical activity (e.g., running wheels) is not standard in animal housing. Moreover, it is debated whether such so-called enrichment of housing is always required, beneficial, or adequate for the outcome of an experiment (Bayne and Würbel, 2012), given that after decades of breeding and selection lab mice in many respects show different behaviors to wild mice (Latham and Mason, 2004).

Recent publications highlight the important role of microbiota in rodents (and humans). Even though SPF animals are the most commonly used rodents in experiments (Fiebiger et al., 2016), gut microbiota are not homogenous (e.g., in composition or bacterial numbers) in such research settings and the extent of this variation has only recently emerged. Microbiota differ between vendors and mouse strains (Hufeldt et al., 2010; Ericsson et al., 2015; Hilbert et al., 2017), different shipments from the same vendor (Hoy et al., 2015), between research animal facilities (Rausch et al., 2016) and even between rooms in the same breeding facility (Rogers et al., 2014). Determining factors for gut microbiota differences under SPF housing conditions without experimental perturbations have been analyzed, and apart from vendor, the fodder and treatment thereof is important (Rausch et al., 2016). Therefore, housing conditions strongly influence mouse microbiota. Data also suggest a general difference between inbred lab mice and wild mice in that the proportion of *Firmicutes* vs. *Bacteroides* vary, with wild rodents being dominated by *Firmicutes* and vice versa (Weldon et al., 2015).

## Misleading results due to inappropriate analysis or an inappropriate model

“If you have cancer and you are a mouse, we can take good care of you” (Kolata, 1998). This famous sentence from Judah Folkman (the “father” of tumor angiogenesis) makes this point: any model - animal or even mathematical - only returns the output it is capable of producing. A translational mouse model that lacks human feature X will never give a response in X, no matter how important that particular feature is in the context of a human disease. While the mouse model has been very successful for understanding the general principles of the mammalian immune system and infectious disease (e.g., Buer and Balling, 2003), it is important to be aware of, and acknowledge, the intrinsic benefits and limitations in any model chosen for a specific experiment. However, before we focus on biological confounders we want to consider that failures in the transition from preclinical studies to humans may also be due to poorly designed or performed studies (see Couzin-Frankel, 2013; Justice and Dhillon, 2016). To illustrate that problems of very different character can challenge the suitability of translational rodent models, we will first discuss two past examples from different disciplines (sepsis and immunology) that caused vibrant discussions in the scientific community and were subsequently analyzed in great detail. They can thus provide valuable insights of general importance for scientists with different research interests. In the section Non-genetic Confounders in Rodent Infections with Protozoan Parasites we will then turn to confounders in translational rodent models of infections with protozoan parasites.

### Analysis, re-analysis and meta-analysis — three studies and three conclusions

Due to its importance for human health, research on sepsis in mouse models is heavily funded, but its translational success has so far been disappointing (van der Worp et al., 2010). Few papers in biomedical research have therefore raised such an excitement and storm of replies and counter-replies as the Seok et al. (2013) study on sepsis. They analyzed transcriptomic data from various mouse models of human inflammatory diseases, and human samples in particular from septic shock, and concluded that “genomic responses in mouse models poorly mimic human inflammatory diseases” (Seok et al., 2013). However, re-analysis of the very same data subsequently concluded the opposite, and these authors simply replaced “poorly” with “greatly” in the article's title of their reply (Takao and Miyakawa, 2015). This discussion is still ongoing, with a recent paper (Weidner et al., 2016) pointing out that the data from Seok et al. *per se* are good enough to compare the transcriptional responses of certain (but not all) mouse models to humans, but that the analytical tools used in the two first papers were inappropriate. The authors' conclusions were that gene set enrichment analysis (GSEA) is more appropriate than gene-to-gene comparisons, which require setting an arbitrary threshold for the determination of differentially expressed genes (as opposed to identification by statistical means). Those tools were used differently by various authors for re-analyses of the original data sets, thus leading to opposite conclusions (Seok et al., 2013; Shay et al., 2013; Takao and Miyakawa, 2015; Warren et al., 2015). A further level of complexity that makes conclusions derived from transcriptomic comparisons challenging is that for many genes there is no correlation between mRNA levels and protein quantities (see section Transcriptomes Do Not Necessarily Predict Protein Levels). Although transcriptome analysis is a fairly easily accessible and promising technique, these examples illustrate that such relatively young tools require close evaluation of the entire work-flow. Understanding and considering the physics or chemistry behind the method and to critically assess appropriate analysis methods is a community task when new scientific methods are being established.

### The CD28 superagonist antibody “disaster”

It is well known that substantial differences between the mouse and human immune systems exist (Mestas and Hughes, 2004; Zschaler et al., 2014; Sellers, 2017) and that they need to be considered when using mice as preclinical models of human disease (e.g., Beura et al., 2016). In 2006, a small human phase I clinical trial aimed at alleviating rheumatoid arthritis tested a humanized monoclonal antibody, TGN1412, directed against the human T cell receptor CD28. However, instead of improving the autoimmune condition, it resulted in devastating consequences (reviewed by Hunig, 2016). It was anticipated from laboratory mouse studies that injection of the antibody would result in the preferential production of regulatory T cells, followed by a downregulation of active T cells. However, all six volunteers had to be hospitalized and at least four of them suffered multiple organ dysfunctions. TGN1412 had caused an immediate “cytokine storm” in these patients due to substantial TNF-α release, followed by dramatically increased plasma concentrations of several cytokines. This “cytokine release syndrome” (CRS) was caused by a strong activation of CD4^+^ effector memory T cells, which eventually caused severe tissue damage. But why had preclinical studies in primate models, namely cynomolgus and rhesus monkeys, not indicated any signs of problems? What was unknown in 2006 was that those primates' CD4^+^ effector memory T-cells do not express CD28 whereas humans do (Eastwood et al., 2010). In this particular case, the monkeys were a poor model for humans, despite their phylogenetically close relationship.

And why had the human response not been seen in the numerous rodent experiments performed prior to the trial? Interestingly, later experiments have demonstrated that at least two drastically modified lab mouse models can indeed be good models for the TGN1412 experiments. The first example is linked to the fact that, as is the case for most immunological experiments, mice in the initial studies had been raised and kept under SPF conditions. Thereby, they had no exposure to microbial antigens that would elicit CD4^+^ memory T cells. Thus, CRS was not initiated upon TGN1412 treatment as it was in humans. Consequently, when TGN1412 was later given to non-laboratory “dirty” mammals (including rodents, see section Getting the Rodent Model “Dirty”) exposed to prior environmental microbial stimuli, they experienced similar syndromes as the human volunteers (Eastwood et al., 2010). The second alternative model consists of humanized mice (see section Humanized Mice: Rodents Which Mimic the Human Immune System). A recent study made use of mice which had been reconstituted with human peripheral blood mononuclear cells. Injecting TGN1412 into those animals recapitulated a number of the disastrous immunological outcomes also seen in the initial human trial (Weissmuller et al., 2016). Importantly, the transplanted human cells also included a small amount of effector memory cells. Therefore, both the use of “dirty” and humanized mice better mimicked human biology than the rodent models which were used in the pre-clinical studies (although both these models have gained interest more recently and were, if at all, very new ideas at that time).

## Non-genetic confounders in rodent infections with protozoan parasites

Lab rodent models have been essential for understanding molecular, cellular, and immunological responses; however, most of the variability inherent in natural populations is not captured by them (Pedersen and Babayan, 2011; Beura et al., 2016). Even so, sources of variation which influence experimental outcomes in lab experiments have also been identified in these very models. Reports based on lab experiments have often not accounted for such variability and instead ascribed differences between experimental groups to the aspect under study.

### Microbiota as a general confounder for rodent experiments

Recently, the role of microbiota as a confounder for experimental outcomes in various scientific fields has gained increasing interest (Servick, 2016), largely due to the development of next-generation sequencing and related methods. Common approaches to study microbiota include sequencing fecal content from lab mice and germ-free mice (discussed in detail in Fiebiger et al., 2016), fecal transplants, antibiotics treatment, probiotics, addition of a specific bacterium, and infectious agents. For biomedical research it is noteworthy that microbiota influences host susceptibility to drugs. One example are proteins encoded by drug processing genes, DPGs, which are responsible for uptake, distribution, metabolism, and excretion of xenobiotics such as drugs (Aplenc and Lange, 2004; Klaassen et al., 2011). DPGs in mouse liver display different expression patterns depending on the microbiota status of the animal (Fiebiger et al., 2016). Some authors have linked microbiota differences to subsequent variation in brain activity and changes in social behavior, a concept referred to as the gut-brain axis (e.g., Foster and McVey Neufeld, 2013; Mayer et al., 2015; Gacias et al., 2016) which is proposed to depend on several factors, including the immune system. Variation in microbiota is known to influence both local and systemic immune function by altering the balance of Th1/Th2 cell composition, influencing re-localization of neutrophils, or affecting macrophage polarization (Denny et al., 2016; Lopes et al., 2016). Taken together, it is therefore not surprising that differences in microbiota can have a substantial impact on protozoan parasite infections in the gut and elsewhere. It is also easy to imagine a situation in which genetically modified mice obtained from one breeder or lab and control mice from other sources leads to unintended differences in microbiota composition with resulting influence on the outcome of infection experiments.

### Protozoan infection experiments are influenced by microbiota

One early study pointing to the importance of microbiota for the establishment of a protozoan infection used germ-free mice which were infected with the intestinal parasite *G. duodenalis* (Torres et al., 1992). The authors demonstrated that the microbiota influences the establishment and nature of intestinal infection with regards to severity and parasite reproductive success. A later study showed that female mice with the same genetic background were either susceptible or resistant to *G. duodenalis* infection (Singer and Nash, 2000). Differences were due to the origin (vendor) of the animals, and the same was true for immunodeficient mice. Co-housing led to resistance in all animals, whereas treatment with antibiotics made all animals equally susceptible to intestinal infection. It was therefore concluded that the microbiota determined the outcome of infection. These studies have recently been complemented with more in-depth investigations of the microbial community, showing changes in the amount of microbiota and its composition upon *G. duodenalis* infection in mice (Barash et al., 2016). Hence, not only do microbiota influence infection outcome but the parasite in turn alters the gut microbiota. These studies emphasize the complexity of gastrointestinal parasite infections. Further analysis of microbiota-parasite-host cohabitation will likely reveal interactions such as competition for nutrients or synergies in metabolism.

The complexity of microbiome influences is not limited to gut microbiota. Skin microbiota has also been shown to influence the outcome of cutaneous leishmaniasis in mice. Its causative agent, *L. major*, differently induced skin lesions, edema, and necrosis in germ-free mice compared to SPF mice upon intradermal infection (Naik et al., 2012). Germ-free mice displayed less disease severity, but also reduced levels of IFN-γ and IL-17A from ɑβ T cells in the infected skin area compared to SPF reared animals. By orally administering antibiotics, the gut microbiota, but not skin microbiota, changed without influencing cytokine production. However, introduction of a skin commensal bacterium, *Staphylococcus epidermis*, did rescue IL-17A production in the skin. The authors concluded that local cytokine production was specifically linked to skin microbiota. In a different study, *L. major* infection altered the gut microbiota of infected animals (but differently depending on mouse strain) (Lamour et al., 2015). Infection changed how gut microbiota correlated with systemic functions such as urine metabolites, plasma metabolites, and the immune system. Such findings also highlight that simple correlations between microbiota and protozoan parasites may not be adequate to elucidate the dynamic role of microbiota during infection.

Interestingly, the microbiota does not only affect the site of infection but can also influence how host and parasite interact at other sites. Recent work provided evidence that the severity of malaria infection with rodent *Plasmodium* spp. can also depend on vendor. Differences in disease severity correlate with differences in microbiota composition (Villarino et al., 2016) or bacterial transcription profiles (Stough et al., 2016), demonstrating systemic effects by the microbiota. A study from 2014 also reported a mechanism for such correlations, describing production of anti-*Plasmodium* spp. antibodies in response to gut colonization, specifically by *E. coli* O86:B7 but not by the reference *E. coli* K12 (Yilmaz et al., 2014). These studies demonstrate that infections in specific compartments which are not colonized by commensal bacteria are nevertheless influenced and such effects must be considered in planning experiments and interpreting results.

#### Excursion 1: a reductionist *in vitro* approach using organoids

Protozoa-host interaction studies have largely been restricted to more or less suitable rodent models, cell lines (often cancer-derived), and short-lived primary tissue cultures from biopsy or surgery. Recent advances in stem cell research have paved the way for the development of self-renewing and complex tissue-like culture systems, so-called organoids, which mimic organs in their main functions and structural features (Willyard, 2015). Major advantages include that host-parasite interactions can be investigated in a primary, long-lived, organ-like tissue from the organism of choice, including humans, in real time (Klotz et al., 2012). Organoids have been developed from the gastro-intestinal tract (GIT), including stomach, gut and liver, and also from kidney and brain (Clevers, 2016). Importantly, organoids lack tissue-specific immune cells and in the GIT the microbiota, and therefore complexity is low compared to *in vivo* settings. However, this feature allows the researcher to construct an experimental setup with exactly the desired level of complexity, adding for instance the Mouse Intestinal Bacterial Collection (Lagkouvardos et al., 2016), human microbiota from biopsies, and/or a set of cytokines or immune cells of interest. For elucidating the role of individual actors during a parasitic infection, organoids are promising alternatives to animal models, cell culture systems, and the use of human biopsy material.

### Sex and age

Two long-known factors that influence infection success by parasites are sex and age. The most obvious differences between the sexes are hormones (Roberts et al., 2001; Klein, 2004; Bernin and Lotter, 2014) but X chromosome-linked mutations (van Lunzen and Altfeld, 2014; Garenne, 2015) and sex-specific behavior can also affect the outcome of infectious diseases. A prominent example in protozoan infections is glucose-6-phosphate dehydrogenase (G6PD) deficiency, which protects humans of both sexes to different extents from clinical outcomes of infections with *P. falciparum* (Shah et al., 2016). A previously developed humanized mouse model of G6PD deficiency (Rochford et al., 2013) has recently been used in screening efforts to identify malaria transmission-blocking drugs (Wickham et al., 2016). A second example involves X-linked immunodeficiency in the B-cell responses due to mutations in the Bruton's tyrosine kinase. The mutation causes a sex-specific effect which leads to X-linked agammaglobulinaemia (XLA). Human patients and mice bearing a similar mutation (CBA/N) are more prone to develop chronic giardiasis (Skea and Underdown, 1991; Van der Hilst et al., 2002).

In rodent models of, e.g., *Plasmodium* spp., *Cryptosporidium* spp., and *Leishmania* spp. infection age significantly influences susceptibility (Adam et al., 2003), parasite reproductive success (Rhee et al., 1999), and severity of disease (Muller et al., 2008). In the case of *Cryptosporidium* (Rhee et al., 1999), hamsters displayed age-dependent differences (within the first 2 months of life) in infection persistence measured by time for shedding oocysts, whereas mice did not. The results demonstrate that rodents can be used to study cryptosporidosis, but simultaneously suggest that generalizations of these results to other species are difficult, and translational success is not obvious. For leishmaniasis, recent work showed mouse age-specific differences in the induction of adaptive immunity. Animals were exposed to a vaccine candidate based on genetically modified *L. donovani* and aged mice (~16 months) had a less pronounced adaptive immune response compared to young mice (~2 months) upon *L. major* challenge after vaccination (Bhattacharya et al., 2016). In *Babesia microti* infection of lab mice between the ages of 2 and 18 months (Vannier et al., 2004), one of three strains (DBA/2 mice) mimicked patterns seen in humans in which susceptibility and an inability to clear infection increased with age. The other two strains displayed smaller differences in susceptibility and no change in infection clearance, illustrating possibilities to use rodents as models for human babesiosis, but alerting to possible issues with interpretation and translation of results.

Given these examples it seems obvious to consider age and sex aspects when planning rodent experiments. However, it is not unusual to use only male or only female mice (Flórez-Vargas et al., 2016) based on convenience, local availability, costs, legal issues (more animals required when both sexes are examined; Clayton and Collins, 2014) or research area. In particular in infectious disease research there is a strong bias toward using female mice (Flórez-Vargas et al., 2016). One reason for this is presumably that they are less aggressive and thus cheaper since they can be housed in (experimental) groups in a single cage whereas this is challenging for male mice. Likewise, younger mice are cheaper to obtain since housing cost are lower. Thus, convenience rather than scientific reasoning might influence the choice of sex or age in many studies.

## Models for all purposes — from fixed alleles to complex ecology

Even before the first draft of the mouse genome was published in 2002 (Waterston et al., 2002), scientists were aware of the relative genetic homogeneity of the lab mouse compared to wild mouse populations (Guenet and Bonhomme, 2003). Inbreeding over almost a century fixed alleles in currently available lab mice, which now represent just a fraction of the genetic variability found in nature. Although a desirable feature for some questions, this variability can be of great importance in studies of host-parasite interactions. Genetic variability might be the reason for different host susceptibility together with, e.g., the confounders discussed above (see section Non-genetic Confounders in Rodent Infections with Protozoan Parasites). In many cases allelic variations of a gene involved in immune responses were identified as the cause of infection outcome.

### When immune responses depend on genetics — selected examples

Allelic variation at Lsh and H2 loci is involved in the opposite outcome of the acquired immune response in *L. donovani* infection between e.g., CBA and BALB/c mice (Loeuillet et al., 2016). Another emerging example is the role that the inflammasome has in sensing protozoan infections (reviewed in Zamboni and Lima-Junior, 2015). During *T. gondii* infection in rodents, sequence differences in the pathogen sensor *Nlrp1* accounts for species-specific inflammasome induction - and thus outcome - in lab mice (Ewald et al., 2014) and rat strains (Cirelli et al., 2014). Phenotypic differences between model animals can also be due to polymorphisms in the inflammasome pathway effectors, e.g., IL-18 and IL-1β. A study on a wild, natural population of field voles (*Microtus agrestis*) found associations with polymorphisms of IL-1β, IL-2, and IL-12β and differential susceptibility to pathogen infection (Turner et al., 2011), with the impact on susceptibility being comparable to parameters like sex and body weight. The parasites considered in the study were mostly nematodes, cestodes, and *B. microti*, making the influence of these cytokines' polymorphisms in other protozoan parasite infections a likely scenario.

Recently, polymorphisms in immunity-related GTPases (IRGs) affecting the rodent immune responses to *T. gondii* were described in a study comparing the DNA sequences of several inbred and wild-derived mice (Lilue et al., 2013). Remarkably, the authors showed that while the sequences of all the examined lab mice were highly conserved, genes in the wild-derived mice were extremely diverse, comparable to the diversity of MHC genes. One of those genes, the highly polymorphic *Irgb2-b1* from a wild-derived *M. musculus*, when expressed in C57BL/6 fibroblasts, was sufficient to confer resistance (i.e., prevent cell lysis) to so-called virulent strains of *T. gondii*. While 1–10 parasites of these strains can kill a lab mouse, IRG-polymorphic wild-derived mice are resistant to infection by much higher numbers of the same *T. gondii* strain. Apart from highlighting extensive sequence variability of wild-derived but not classical inbred laboratory strains in these gene loci, this work emphasizes that the definition of virulence is heavily dependent on the animal of choice and that its definition should always be accompanied by stating the experimental conditions. In the following section we move from traditional, genetically homogenous and inbred mice to mice manipulated to resemble aspects of the human immune system, and then to genetic crosses between inbred and wild-derived mice. Lastly, we will turn our attention to “dirty mice” and wild rodents in natural settings.

### Humanized mice: rodents which mimic the human immune system

Designing an immune system with human features within the mouse—generating humanized mice—has recently emerged as an approach to expand the areas where lab mice can be used to model disease. Generation of humanized mice is based on immunodeficient animals (e.g., SCID, Rag2^−/−^) whose innate and adaptive immune systems are severely compromised and the animals are instead characterized by increased survival of transplanted human hematopoietic cells (Kaushansky et al., 2014; Good et al., 2015), which produce a large number of different human immune cells in the mouse. Depending on further needs, these mice can be populated with, for example human red blood cells and/or CD34^+^ hematopoietic stem cells that further give rise to T cells and antigen presenting cells (APC). Recent advances in the development of humanized mice offer the possibility to study human infectious diseases which could previously not be investigated in mice, in the mouse model (Brehm et al., 2013). Even though the use of rodent-infecting *Plasmodium* spp. such as *P. berghei* has greatly contributed to understanding the parasite's biology and general principles of protective immune mechanisms in mammals (Craig et al., 2012), it is promising that human-infecting *P. falciparum* now can be researched in lab mice (Kaushansky et al., 2014). The basic strategy for generating humanized mice, and adaptations of it lead to the generation of mice in which the blood stages of human malaria parasite life cycles could be established (Kaushansky et al., 2014; Good et al., 2015).

More advanced models with engrafted human hepatocytes (FRG-NOD huHep) have been further used to establish the complete development of the pre-erythrocytic liver stage of *P. falciparum* after mosquito bite, including formation of exo-erythrocytic merozoites, subsequently infectious to human red blood cells in the same mouse (Vaughan et al., 2012). Although, the development of the mature sexual stages (gametocytes) that are necessary to complete the parasite life cycle is still inefficient, it seems possible that complete *P. falciparum* (and other human *Plasmodium* spp.) life cycles could be routinely maintained using humanized mice. Recently, such mice were also used to conduct a genetic cross between two *P. falciparum* strains in those animals, something that so far was only possible in non-human primates, including chimpanzees (Vaughan et al., 2015).

These examples and others from several other infectious agents (Ernst, 2016) suggest that humanized mice will continue to contribute to a new repertoire of mouse translational models. Understanding host specificity factors for a given human pathogen is crucial for the design of susceptible humanized mice, and methods to identify such factors are described in detail by Douam et al. (2015). However promising, the establishment of these mice is relatively new and already several limitations are known (described in more detail in Ernst, 2016), which limit the extent to which the models actually mimic the human immune system. For instance, several mouse cytokines differ largely in their sequences between mouse and human, and IL-13 has no effect on human cells. This might explain the low proportions of certain human immune cell types in humanized mice. In addition, signaling and adhesion molecules are different between humans and mice and, importantly, the expression of murine and not human major histocompatibility complexes impairs the function of T cells. Attempts have been made to account for some of these limitations (Ernst, 2016) but so far humanized mice are probably best considered as a promising, but yet developing, tool in translational research.

Even so, host-specificity has limited the choice of model systems for studying protozoan parasites. The *Plasmodium* spp. examples and initial attempts with *L. major i*nfection in humanized mice (Wege et al., 2012) hold promise for future possibilities to investigate also other human-specific protozoan parasite species in lab mice.

### Mixing the known — recombinant inbred strains and the collaborative cross

In order to document an influence of genetic heterogeneity on experimental results, models beyond inbred animals are required (Phifer-Rixey and Nachman, 2015; Chow, 2016). This is also true in translational research, where humans represent a genetically diverse population. When the aim is to find differences across the genome, as in genome-wide association studies (GWAS) where a given phenotype is thought to be linked to genetics, outbred stocks of mice or rats are not a solution since they are “a genetically ill-defined set of laboratory mice that are often used erroneously in toxicology, pharmacology and basic research” (Chia et al., 2005). In addition, their usefulness is limited for practical reasons, e.g., individual phenotypic variability requires larger sample sizes than necessary with inbred strains, or the study will lack statistical power to correlate experimental differences with certain genotypes (see Chia et al., 2005; Festing, 2014). In order to address the problem of limited genetic diversity but avoid the ill-defined genetic composition of wild animals, numerous mouse collections besides the RIS (see section The Mice We Use in Experiments — Who Are They and How Do They Live) have been established.

**Figure 3:**
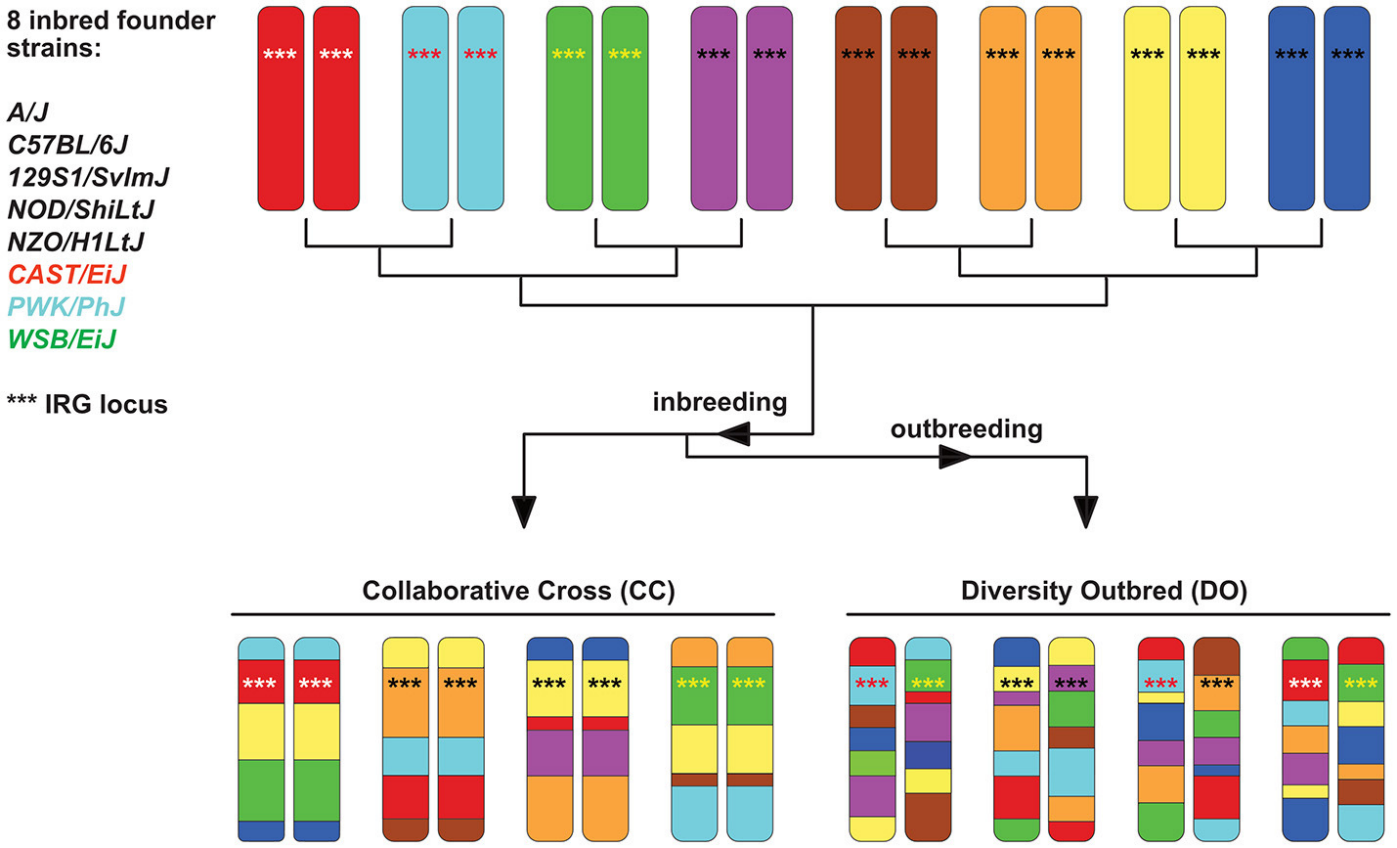
Scheme explaining the principle of Collaborative Cross (CC) and Diversity Outbred (DO) sets (based on Chick et al., 2016). Initially, they all derived from 8 inbred founder strains. Chromosome 11 is given as an example, with the IRG locus (see section When immune responses depend on genetics — selected examples) indicated by ^***^. The locus is highly homogenous in 5 of the 8 founder strains (black ^***^) but highly polymorphic in the 3 wild-derived strains CAST, PWK, and WSB, indicated by differently colored ^***^ in the chromosomes.

The concept for producing RIS sets has been extended by the Complex Traits Consortium to produce more genetically variable sets (Chia et al., 2005). These are known as Collaborative Crosses (CC) and are based on a set of 8 defined and sequenced founder strains, including three wild-derived strains of *Mus* and five traditional inbred strains. Although the set of strains is genetically diverse, each CC strain is at least 90% homogenous and hence genetically well-defined. The CCs were designed specifically for complex trait analysis (Churchill et al., 2004; Threadgill and Churchill, 2012) and the derived Diversity Outbred (DO) population (Churchill et al., 2012) has resulted in an even more genetically diverse mouse population (see Figure 3). Other derivatives of CC's concept exist, like the Heterogeneous Stock mice (Valdar et al., 2006) or the Hybrid Mouse Diversity Panel (Bennett et al., 2010). Besides GWAS studies, which map determinants for non-infectious diseases, CC animals have recently been used to map susceptibility or pathogenesis determinants in bacterial and viral infection models (Durrant et al., 2011; Ferris et al., 2013; Rasmussen et al., 2014; Vered et al., 2014; Gralinski et al., 2015; Lore et al., 2015; Smith et al., 2016). However, no data for parasite infections have been reported so far, and their large potential for exploring how host genotype influences infections needs to be explored in the future. Nevertheless, CC and DO mice also have limitations (Phifer-Rixey and Nachman, 2015). They are all derived from subspecies, which limits the genetic variation and may cause partial hybrid sterility in crosses. This, in turn, might have resulted in the elimination of genetic variation at these genomic loci. However, it is expected that further crosses will improve these models on the genetic level.

Yet, these mice will never approach the genetic diversity found in the human population or wild animal populations. In addition, individuals in natural populations encounter seasonal and spatial variability in the environment, as well as differences in climate and food availability. Wild animals are also exposed to and infected with a vast array of parasites and other pathogens, harbor different microbiota, and individuals vary in their demography, behavior and genetic composition. While it is possible to add key elements of natural variation into the above described rodent model systems, there is an increasing interest in moving beyond the controlled laboratory setting to a more realistic scenario.

#### Excursion 2: metabolic disease in lab mice and humans — is ecological complexity better than SPF facilities?

Around 25% of the world population has a metabolic syndrome, defined by the International Diabetes Federation as either diabetes / prediabetes, abdominal obesity, high cholesterol or high blood pressure. Animal studies provide important information on these conditions (Bäckhed et al., 2004; Turnbaugh et al., 2006). The role of microbiota has gained interest also in this field because of potential new treatments which can manipulate the microbiome community and function (Borody and Khoruts, 2012). Of interest here are the large numbers of studies conducted in rodents which demonstrate, for instance, alterations in body weight and insulin sensitivity which correlate with changes in microbiota upon antibiotics treatment in these model rodents (Bäckhed et al., 2004; Turnbaugh et al., 2006). Although similar data also exists from human studies and therefore support that translation of results from rodent models to humans in this case is possible, a recent contribution to this topic questions the rodent model to mimic metabolic disease and microbiota correlations in humans. In a study performed on 57 overweight and obese adult men, the systemic effects of two antibiotic treatments compared to placebo were investigated (Reijnders et al., 2016). Changes in microbiota composition (detected by 16S rRNA microarray analysis) were observed for specific antibiotics against gram-positive bacteria, but no differences were seen with the broad-spectrum antibiotic. On most other readouts, the authors did not see significant responses to antibiotics treatment. Hence, changes in microbiota composition did not correlate with changes in systemic functions in humans (e.g., insulin sensitivity, energy metabolism and gut permeability; Reijnders et al., 2016), which is in contrast with rodent data (Bäckhed et al., 2004; Turnbaugh et al., 2006). Reijnders et al. (2016) discuss hypotheses for these discrepancies, mentioning treatment duration and the method of antibiotic intake (capsules or in water). In addition, a possibly important difference between the described rodent studies and the human study is the fact that humans are a “wild” population. The genetic heterogeneity and environmental influences in the human population of 57 men are indeed different from the SPF bred rodents. The effects of both previous and current microbe colonization and/or infections do influence immune responses in an individual and constitute an important difference between wild and controlled laboratory populations (reviewed in Tao and Reese, 2017). Possibly, the use of a “dirty” or wild rodent population would be a more suitable choice when the aim is to investigate correlations in a highly complex biological system.

### Getting the rodent model “dirty”

In the continuum of approaches that can be employed to better understand protozoan parasite infection and immunity, “dirty” animals taken from the wild or laboratory animals exposed to wild cage-mates have emerged as a promising model (Maizels and Nussey, 2013). Arguing for such translational models, recent results demonstrate that inbred mice reared in SPF conditions have the immunological phenotype of neonatal humans, lacking effector-differentiated and mucosally distributed memory T cells (Beura et al., 2016). In contrast, “dirty” *M. musculus* brought in from either a pet shop or from feral barn populations had immune responses more similar to adult humans, with high levels of memory CD8^+^ cells, likely due to diverse microbial exposure and infection. These changes in both the innate and adaptive immune cellular responses and immune gene expression could also be recapitulated by co-housing previously SPF inbred mice with pet shop mice. While about 20% of the SPF mice died due to microbial infection, the immune response of those that survived also resembled adult humans within 4–8 weeks, with effector-differentiated and mucosal memory T cells. In addition, within that short time frame, co-housed mice responded similarly to the wild-caught pet shop mice in terms of infection, such that they were significantly more resistant, amongst other pathogens, to challenge with the cerebral malaria model *P. berghei* (Beura et al., 2016). Another research group has aimed to make their inbred laboratory mouse strains “dirty” by giving them sequential infection with mouse herpes virus, influenza and an intestinal helminth in order to test how this more natural pattern of exposure to pathogens may affect immune variation and expression after vaccination (Reese et al., 2016). They found that co-infected mice had different immune gene signatures, cytokine expression and antibody levels in the blood both before and after yellow fever virus vaccination compared with their SPF lab mice controls. These expression patterns resembled those of pet store-raised mice. While getting the traditional sterile laboratory mouse models dirty may pose logistical challenges, such results should encourage researchers to revisit abandoned vaccine candidates as well as to establish different routines for testing new ones.

### Benefits of using wild mice

Studies on dirty mice with the benefits described above still lack other aspects of natural variation that are important (Pedersen and Babayan, 2011). Thus, there is a need for wild model organisms that permit robust studies of the individual and environmental variation inherent in natural populations (including humans). Populations of wild mice vary in many of the same ways as humans (e.g., age, sex, condition, resources, parasite exposure, infection/co-infection, genetics, etc.), yet can provide a tractable, experimental system to test the importance of natural variability on infection, immunity and disease control. There are several key epidemiological features in wild mouse populations that closely resemble human infection dynamics, such as having great variation in infection probability, burdens and disease severity across individuals. Moreover, wild mice are commonly found chronically infected with parasites, suggesting either a high frequency of re-infection, long-lasting infections, or both (Pedersen and Babayan, 2011; Knowles et al., 2013).

One approach to start a research program on wild rodents is to study the traditional laboratory mouse species (*M. musculus*) in the wild (Potter et al., 1986; Viney et al., 2015). Abolins et al. (2011) found that the immune function of wild-caught *M. musculus* was significantly greater than lab-reared C57BL/6 mice, such that after immunization with a novel antigen wild-caught mice had higher concentrations of total IgG and IgE, produced higher and more avid concentrations of antigen-specific IgG, and had greater activation of T helper cells, macrophages and dendritic cells than lab-reared mice. While wild *M. musculus* offer a great parallel to lab-reared mice and can serve as comparisons for protozoan infection in the laboratory, many studies do not exhaustively sample for ectoparasites and protozoans and their true infection status is not well described. Commonly, wild *M. musculus* are reported to be infected with mainly ectoparasites and a few nematodes (mostly *Syphacia spp*. pinworms; e.g., Weldon et al., 2015); however there are records of natural infections with *Giardia muris, Spironucleus muris*, and *Encephalitozoon cuniculi* (Baker, 1998), *Eimeria* spp. (Ball and Lewis, 1984), *Cryptosporidium* spp. (Backhans et al., 2013), and *T. gondii* (Kijlstra et al., 2008).

### Rodents beyond wild mice are natural hosts of a wide variety of protozoa

Beyond *M. musculus*, there are several well-studied wild rodents that are both commonly infected with protozoan parasites and also offer tractable wild model systems for both longitudinal and experimental studies of infection and immunity. In North America, much work has focused on white-footed mice and deer mice (*Peromyscus leucopus* and *P. maniculatus*), both because they are very abundant and widespread, but also because they are competent reservoirs of important emerging zoonotic pathogens (e.g., Hantavirus and *Borrelia* spp.; Bedford and Hoekstra, 2015). In Europe, wood mice (*Apodemus sylvaticu*s; e.g., Knowles et al., 2013), yellow-necked mice (*A. flavicollis*; e.g., Ferrari et al., 2004), bank voles (*Myodes glareolus*; e.g., Withenshaw et al., 2016), and field voles (*M. agrestis;* Smith et al., 2005; Turner et al., 2014) have been commonly studied as models for wild host-pathogen interactions and are all regularly infected with protozoan parasites. For example, wild populations of *A. sylvaticus* in the United Kingdom have been found to be infected with *C. parvum, C. muris* (Chalmers et al., 1997); > five species of *Eimeria* (Ball and Lewis, 1984; Higgs and Nowell, 2000); *Babesia* sp. and *Hepatozoon* sp. (Turner, 1986); two species of *Trypanosoma* (Noyes et al., 2002), *Frenkelia microti* (Svobodova et al., 2004) and *T. gondii* (Jackson and Siim, 1986). It is very likely that this list is a far from exhaustive.

The benefits of using wild rodent-parasite models to better understand protozoan infection dynamics include the ability to: (i) conduct longitudinal field experiments which follow marked individuals throughout their lives while measuring infection status, physiological and demographic metrics (Knowles et al., 2013; Pedersen and Antonovics, 2013; Turner et al., 2014), and crucially (ii) test the efficacy of disease control interventions at the individual and population level in an ecologically relevant environment (Knowles et al., 2013; Pedersen and Antonovics, 2013). For example, in a population of wild field voles (*M. agrestis*) the researchers repeatedly treated one population with a standard insecticide to reduce the prevalence of fleas, and in turn, found that this reduced the prevalence of vector-transmitted *Trypanosoma* spp. by ~33% (Smith et al., 2005). In addition, in experimental field studies of both *P. maniculatus* and *P. leucopus* in the US, and *A. sylvaticus* in the UK, anthelmintic treatment was used to reduce nematode burdens within specific, marked animals. The treatment was found to unexpectedly increase the prevalence and/or intensity of co-infecting *Eimeria* spp. suggesting strong antagonistic within-host interactions between a worm and a protozoon (Knowles et al., 2013; Pedersen and Antonovics, 2013).

Research on wild rodents benefit from the extensive immunological toolbox developed in lab mice (Pedersen and Babayan, 2011). In wild populations of *A. sylvaticus*, innate immune responsiveness, as measured by splenocyte tumor necrosis factor responses to toll-like receptor (TLR) agonists, was found to correlate positively with *Eimeria* spp. fecal oocysts counts, most strongly with receptors TLR7 and TLR9 (Jackson et al., 2009). More recently, the availability of genomes for wild rodents has enabled the ability to measure immunological expression in wild rodent populations. A recent investigation of wild field voles measured expression of a wide range of innate and adaptive responses by cultured and stimulated splenocytes. Importantly, repeated measures from peripheral blood samples of IFN-y, Gata3 and IL-10 expression enabled the authors to test for correlations with specific parasite infections (Jackson et al., 2014). Taken together, wild rodents reach large sample sizes, can be repeatedly recaptured using live traps, marked and followed before and after interventions, and are commonly infected with protozoan parasites. Studying the dynamics of protozoan infections in wild rodents is a valuable resource for expanding our knowledge in infection biology and might thus be a useful addition for translational research on human protozoan infections.

### A case for going wild: do *T. gondii*-induced behavioral changes exist in natural habitats?

How relevant are findings which suggest parasite influences on lab mouse behavior when performed in lab environments? The so-called “manipulation hypothesis” of a *T. gondii* infection in rodents suggests that infection leads to subsequent changes in the animal's behavior, with one consequence being that they lose their fear for feline odor (e.g., fur or urine). Cats and other felids are the only definite hosts where sexual reproduction of *T. gondii* can take place. Therefore, at first sight it makes sense that such “manipulated” infected rodents would experience more fatal encounters with a cat than non-infected ones, thereby increase the chance for *T. gondii* to sexually reproduce with another strain from a second subsequent infected prey.

The advantage or necessity of this scenario for parasite sexual reproduction in the wild has been called into question (Worth et al., 2013), but here we focus on the fact that all reported experiments were done exclusively in lab animals (Worth et al., 2014). At first sight this might not seem problematic since *M. musculus* and *T. gondii* naturally occur together. However, it is well known, but not necessarily well appreciated, that behavioral studies of rodents can be influenced by the methods used, housing conditions, genetic background and whether they are lab or wild-derived animals (Wolff, 2003; Beckers et al., 2009; Fonio et al., 2012; Chalfin et al., 2014; Newman et al., 2015). Even differences in the microbiota can have profound effects (Hsiao et al., 2013; see section Microbiota as a General Confounder for Rodent Experiments). Moreover, lab mice have been selected for decades for docile behavior, while wild mice show anxious behavior under natural conditions (Latham and Mason, 2004; Yoshiki and Moriwaki, 2006; Fonio et al., 2012; Chalfin et al., 2014).

How well does the manipulation hypothesis apply to the natural situation of predator (cat) and prey (*T. gondii*-infected mouse or rat)? Ecological observations might explain some of the observed discrepancies, i.e., no behavioral differences were found in one study but were indeed found in another (Worth et al., 2014). Several studies indicate that predation risk of wild or wild-derived small rodents depends more on habitat characteristics (e.g., ability to hide) than on whether the rodent senses a present predator by its odor or even by its physical presence (Orrock, 2004; Powell and Banks, 2004; Verdolin, 2006). Aversive responses to predator odor can also differ dramatically between individual lab rats (Hogg and File, 1994) and, importantly, between lab mice and wild mice (Coulston et al., 1993; Hebb et al., 2004). Thus, experiments with “fearless” lab mice in non-natural terrains may not accurately reflect the behavioral changes induced by a parasite like *T. gondii* under natural conditions.

## Experimental challenges and available resources for non-traditional rodent models

Having provided a number of reasons for considering wild rodents as alternatives, we will briefly address the experimental challenges. Approaching studies on non-model rodents is demanding but the toolbox has improved significantly compared to a decade ago (Pedersen and Babayan, 2011; Zimmerman et al., 2014). Method development in the fields of genomics, transcriptomics and proteomics together with increasing affordability provide better prerequisites for research on non-model alternatives (Jackson, 2015). Next-generation sequencing has led to constantly expanding genomic data. This is demonstrated by the nearly 4,000 eukaryotic genomes available on NCBI Genome (February, 2017) as compared to around 650 in 2013 (Ellegren, 2014).

### Database resources for wild rodent genomes

**Figure 4:**
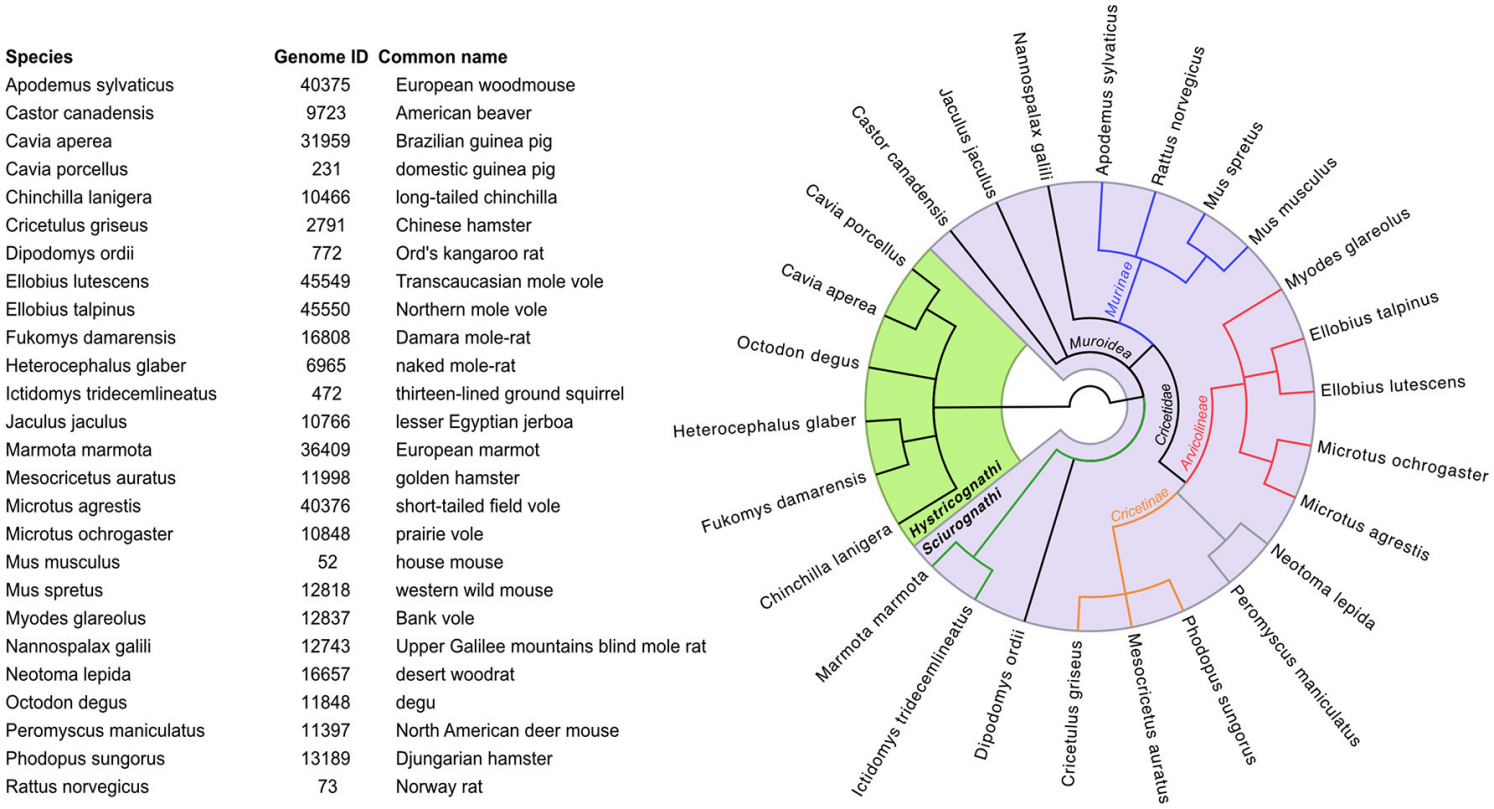
Names of rodents (with links in Table S2 to their available genome sequences at NCBI), together with their phylogenetic relationship (tree computed at https://www.ncbi.nlm.nih.gov/Taxonomy/CommonTree/wwwcmt.cgi).

Any resources available for lab mice are to varying extents useful starting-points for work on wild rodents. The Mouse Genomes Project is the biggest collection of genomic data on rodents (Table S1). Currently it consists of whole-genome assemblages and strain-specific gene annotations of 16 inbred and wild-derived mouse strains. A goal of this project is the classification of sequence variations between common laboratory strains compared to the reference strain C57BL/6J (Adams et al., 2015; Doran et al., 2016). All sequence reads, variants and assemblages can be useful references for highly recombinant outbred strains (Nicod et al., 2016) or wild rodent genomes. There are also increasing numbers of genomes and/or transcriptomes available for wild rodents (e.g., *A. sylvaticus, M. glareolus*, and *M. agrestis*; see Figure 4), with most of them being “work-in-progress” considering assemblage status and annotations (for details see Table S1 and links therein). The quality and coverage of these genomes vary and there is for instance no clear definition of a required coverage for referring to DNA sequences as a “genome” (Ellegren, 2014). However, they do provide a good source for homology searches for a gene-of-interest, primer design for PCR applications etc. Naturally, purification of DNA, RNA or proteins and functional PCR protocols may require protocol optimization when applied to new species but otherwise follow established schemes. Some database resources and other initiatives to promote such development are discussed below and summarized in Table S1.

While reference genomes are not a prerequisite for some studies they are, for instance, indispensable as a template in quantitative gene expression studies with high-throughput sequencing of RNA (RNA-seq; Vijay et al., 2013). However, bioinformatics pipelines are often developed for established model organisms and may require considerable adjustments for non-model organisms (Ekblom and Wolf, 2014). Although such limitations may hamper the speed of omics applications to non-model organisms, genomes of non-model rodents will serve as excellent resources for developing species-specific tools to measure, for example, expression of immunological responses to infection. Readers that are interested in considering genome sequencing for their own non-model organism are referred to a recent step-by-step introduction of the required workflows (Ekblom and Wolf, 2014) and to the Generic Model Organism Database (GMOD) initiative, which provides software tools and data models for subsequent representation of their annotated genomes and curated data of their model organism (O'Connor et al., 2008).

### Transcriptomes do not necessarily predict protein levels

It should be emphasized that genomic and transcriptomic data, as valuable as they are, provide only indirect means with regards to proteomic output in response to infection. In general, the relationship between the concentration of a given transcript and its encoded protein(s) is difficult to predict just by RNA-seq or qPCR data (Liu et al., 2016). For example, Chick et al. (2016) used the currently most sensitive technology for abundance determination of both transcripts and proteins and applied them to CC and DO mice (see section Humanized Mice: Rodents Which Mimic the Human Immune System and Figure 3). They showed that for many genes the levels of the corresponding protein varied substantially in genetically divergent mice. Sex also influenced protein amounts within a given species (Chick et al., 2016). These recent data emphasize the importance of quantitative proteomic measures in general to complement or validate transcriptomic data, but also highlight that genetic diversity within mice influences the results.

### Antibodies, cytokines, and protein quantification

Antibody-based assays are still the cornerstone for qualitative and quantitative determination of immunological parameters like chemokines or cytokines but also other proteins of interest. Numerous well-defined and evaluated reagents exist for lab mice and rats but their usefulness for wild rodents with respect to cross-reactivity is largely unexplored and presumably quite low in many cases. The same applies to immunological effector molecules like cytokines, for which IFN-γ is a good example. IFN-γ is known to be highly species-specific, which made the production of a recombinant protein active with *M. glareolus* or *Microtus* spp. cell lines a prerequisite (Torelli et al, in preparation). Starting from genomic sequences and going to the purification of active recombinant protein from *E. coli* required less than half a year and will now provide the scientific community with this important cytokine.

Developing antibodies that (cross)react with wild rodents is certainly much more time and resource-consuming, but feasible. An alternative could be parallel (or selective) reaction monitoring (PRM, SRM) which are mass spectrometry-based methods that quantify unique, specific peptide sequences of a given protein (Rauniyar, 2015; Bourmaud et al., 2016). The method was recently used to quantify several cytokines and chemokines from human cells (Muqaku et al., 2015). The appealing aspect of this admittedly demanding method lies in the fact that by carefully selecting peptide sequences conserved between rodent species, they could be used across those species at relatively low cost, once established (Hüttenhain et al., 2009). Ideally, it could thus be regarded as a community effort. A database with corresponding peptides from human and mouse proteomes does exist (see Table S1) and constitutes a useful starting point (Peptide Atlas; Deutsch et al., 2008).

Given the availability of published genomic and transcriptomic data of wild rodent species (Table S2), work similar to the studies mentioned will hopefully expand the current toolbox for non-model rodents in the near future. A dedicated web site with information on such shared resources, but also on cross-reacting reagents such as polyclonal or monoclonal antibodies or commercial cytokines and other proteins tested in non-model rodents, although not yet existing, would greatly boost the interest and ease of use of non-model organisms in future studies.

## Concluding remarks

We are convinced that rodents will continue to be important translational models for research on protozoan parasites, given that appropriate considerations are made during experimental design. By providing some examples where translation from rodent disease models to human medicine has failed, and, more importantly, by pointing at identified reasons for inconclusive or misleading data, we wish to inspire readers to consider more than the most convenient model for future experiments. Making use of rich database-resources that are available for investigating, e.g., expected phenotypes of mice, will aid in this respect. We also hope that readers are encouraged to consider and control for various confounders such as microbiota influences and housing conditions in their experimental designs. While wild models pose some challenges, we have pointed out that these rodents possess distinct advantages with regards to genetic variability and environmental exposures that can reflect immunological responses to parasites in humans more adequately than current lab models. The increasing availability of genome and transcriptome datasets as well as improved methods for quantitative proteomics already show their impact on wild infection biology.

## Author contributions

TE, FT, CK, AP, and FS contributed to the text and approved its final version.

### Conflict of interest statement

The authors declare that the research was conducted in the absence of any commercial or financial relationships that could be construed as a potential conflict of interest.
